# The integration of graphene into microelectronic devices

**DOI:** 10.3762/bjnano.8.107

**Published:** 2017-05-15

**Authors:** Guenther Ruhl, Sebastian Wittmann, Matthias Koenig, Daniel Neumaier

**Affiliations:** 1 Infineon Technologies AG, Wernerwerkstrasse 2, 93049 Regensburg, Germany; 2 Nanochem CoE, OTH Regensburg, Seybothstrasse 2, 93053 Regensburg, Germany; 3 University of Siegen, Department of Electrical Engineering and Computer Science, Hölderlinstr. 3, 57076 Siegen, Germany; 4 AMO GmbH, Otto-Blumenthal-Strasse 25, 52074 Aachen, Germany

**Keywords:** contacts, deposition, encapsulation, graphene, process integration

## Abstract

Since 2004 the field of graphene research has attracted increasing interest worldwide. Especially the integration of graphene into microelectronic devices has the potential for numerous applications. Therefore, we summarize the current knowledge on this aspect. Surveys show that considerable progress was made in the field of graphene synthesis. However, the central issue consists of the availability of techniques suitable for production for the deposition of graphene on dielectric substrates. Besides, the encapsulation of graphene for further processing while maintaining its properties poses a challenge. Regarding the graphene/metal contact intensive research was done and recently substantial advancements were made towards contact resistances applicable for electronic devices. Generally speaking the crucial issues for graphene integration are identified today and the corresponding research tasks can be clearly defined.

## Introduction

Since the discovery of the electronic properties of graphene in 2004 [1] a lot of possible applications have been envisioned. Especially in the field of microelectronics graphene holds the promise for faster, more sensitive and even completely novel devices [2]. So, graphene was claimed as one possible material to overcome the foreseeable limits of silicon technology. The fascinating properties of graphene, such as extremely high charge carrier mobility of more than 200,000 cm^2^·V^−1^·s^−1^ [3], was consistently shown in academic research. For instance, a sheet of high-quality graphene sandwiched between two exfoliated single-crystalline hexagonal boron nitride (h-BN) sheets shows a charge carrier mobility of 200,000 cm^2^·V^−1^·s^−1^ [4], which is about 300 times higher than that of silicon. However, if graphene is integrated in real-world devices with the constraints of manufacturability, the properties of graphene and its devices dramatically degrade. The integration of graphene demands a paradigm shift for material integration concepts, in conventional semiconductors the materials properties are mostly determined by the bulk of the semiconductor, and the surface can be cleaned and modified without significantly affecting the materials properties. On the other hand graphene and other 2D materials consist only of surface and every surface modification changes the materials properties. This property allows the use of graphene as an environmental sensor, but is detrimental to fabricating stable devices. In the following review we highlighted the most crucial aspects of the integration of graphene into complementary metal-oxide semiconductor (CMOS) compatible electronic devices.

## Review

### 1 Deposition of graphene

Graphene films can synthesized in numerous ways, such as mechanical exfoliation, liquid-phase exfoliation, assembly of tailored precursor molecules, epitaxy on silicon carbide or chemical vapor deposition (CVD) on catalytic metals [5]. Besides meeting the requirements of film quality and cost, the scalability to 200 or 300 mm wafer sizes is crucial for being suitable for industrial production. Currently, the highest-quality graphene synthesis method that fulfills these constraints is graphene CVD on copper substrates [4,6]. However, for building electronic devices graphene typically has to be placed on an insulating substrate. Hence the graphene films have to be transferred from their growth substrate to the device substrate. In the past several methods have been proposed [7], which can be grouped into the following categories.

#### 1.1 Ex situ transfer

The CVD growth substrate can either be a copper foil, which is most commonly used, or a Cu film deposited by physical vapor deposition (PVD) on a silicon wafer substrate with a diffusion barrier between the silicon substrate and the Cu film. The first approach allows for the production of very large graphene films on a polymer carrier foil even in a roll-to-roll system [8–9]. The second approach is obviously more wafer-level compatible, but at deposition temperatures around 1000 °C, even with diffusion barrier, it suffers from the diffusion of Si from the substrate towards the Cu surface generating holes in the graphene film. Decreasing the deposition temperature to below 800 °C can improve the defect level but cannot completely eliminate it [10]. Also the transfer onto a dielectric substrate wafer for device fabrication in a wafer bonder tool has not been established yet. Both techniques have in common that the strongly different thermal expansion coefficients of graphene (−6 × 10^−6^ K^−1^) [11–12] and the copper substrate (16.5 × 10^−6^ K^−1^) [13] typically lead to wrinkles and cracks in the graphene film. Lowering the deposition temperature would be also beneficial here. Several approaches have been reported [14–18], but have not been shown yet to yield an acceptable graphene quality.

**Figure 1:**
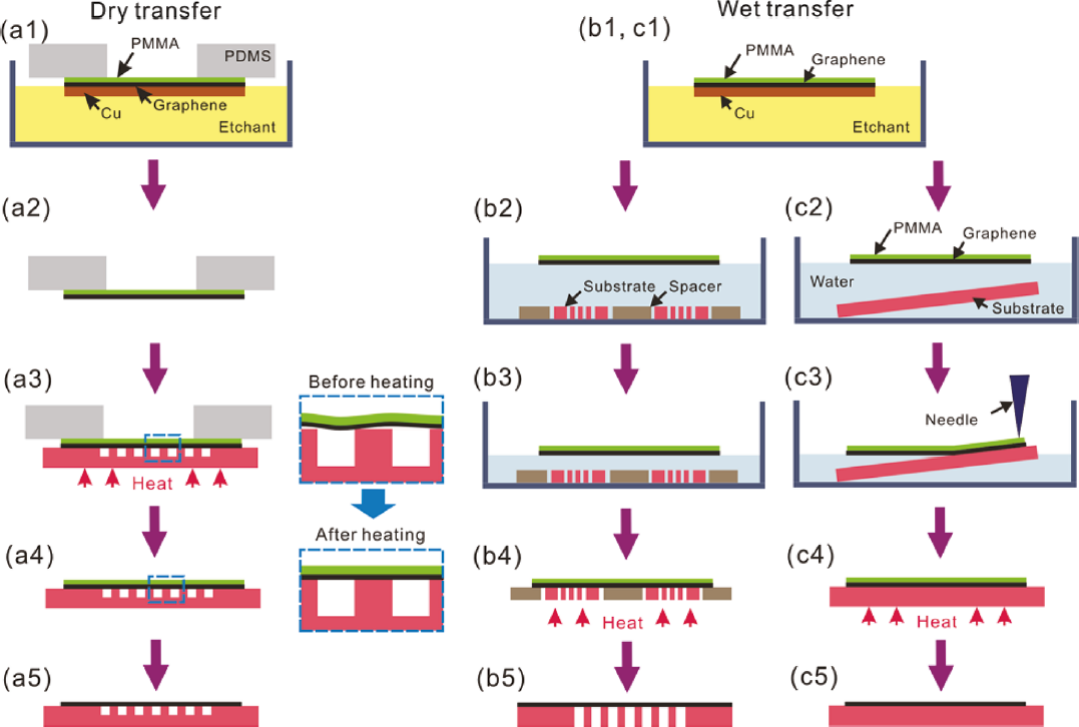
Schematic illustration of dry and wet transfer processes. (a) Dry transfer onto shallow depressions. Wet transfer onto (b) perforated substrates and (c) flat substrates. The boxes with dashed lines in (a3) and (a4) show magnified views. Reprinted with permission from [19], copyright 2011 American Chemical Society.

In history, the first transfer technique for graphene films from the CVD growth substrate was the ex situ transfer by reinforcing the graphene layer with a polymer film, e.g., poly(methyl methacrylate) (PMMA), and etching off the Cu growth substrate. There are several options for subsequent graphene deposition onto the final substrates discussed in [19] (Figure 1).

Focusing on wafer-level processes, the metal underetch times are rather long, even when the graphene film is patterned to provide distributed access for the etch medium. A technique that copes with this problem is the so-called “bubble transfer”, which uses the generation of electrochemically generated hydrogen bubbles at the graphene–metal interface to release the polymer-supported graphene film without the need of lengthy underetching [20–21]. Nevertheless, defects generated by the hydrogen bubbles have to be considered.

After release of the polymer-reinforced graphene film from the growth substrate the graphene is contacted with the dielectric device substrate, either using a liquid medium (“wet transfer”) [19] or a flexible stamp [19] (“dry transfer”). Due to its high adhesion energy on hydrophilic substrates such as SiO_2_ (450 mJ/m^2^) [22] supported by the formation of a water layer [23] graphene attaches to the substrate and, after drying, the supporting polymer is removed by using a solvent. However, as water molecules should completely be removed from the graphene–substrate interface in order to reduce doping effects, the use of hydrophobic substrates is of high interest.

#### 1.2 In situ transfer

The ex situ transfer approach suffers from the potential introduction of defects between the graphene film and the device substrate as well as from the generation of cracks in the graphene film itself [24]. For this reason, a preferable technique would be the graphene deposition on the final device substrate itself. As current methods are using a catalytic metal layer, one approach is the in situ removal of the metal layer between graphene and dielectric substrate by wet etching. In order to attach the graphene film to the substrate an additional adhesion mechanism has to be involved. One approach is the use of capillary forces introduced by gas bubbles released from the substrate during Cu wet etch [25]. The resulting graphene quality is usually better than produced by ex situ transfer. However, this approach suffers from a limited Cu film thickness, which can lead to voids in the catalytic growth layer due to Cu evaporation and stress migration during CVD growth. As already mentioned above the long underetch time causes problems.

#### 1.3 Direct deposition

**Figure 2:**
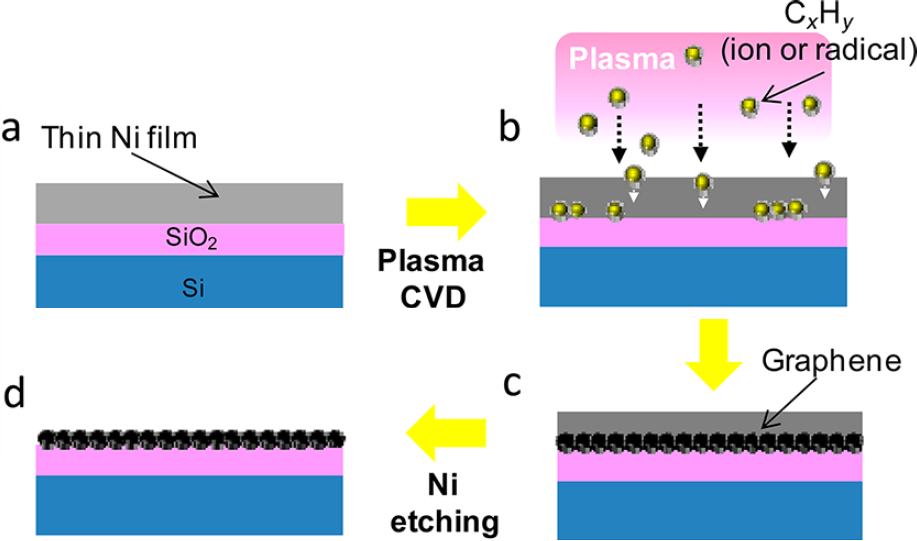
Schematic illustration of the basic approach for the method of direct growth of graphene on a SiO_2_ surface. (a) A thin Ni film is deposited on a SiO_2_/Si substrate. (b) Plasma CVD is performed. (c) Carbon atoms diffuse into the Ni film, and graphene is preferentially grown along the interface between the Ni and SiO_2_ layers. (d) Graphene on a SiO_2_/Si substrate is realized by removing the Ni film using a chemical etching technique. Reprinted with permission from [29], copyright 2012 American Chemical Society.

The ideal procedure includes the direct deposition of graphene on the dielectric device substrate without a catalytic metal layer in between. Two concepts can be characterized by the type of metal removal. The first approach is based on the removal of the metal layer during the CVD process by evaporation [26] or agglomeration [27]. However, the evaporation process lowers the graphene quality. The other approach is the implementation of a dissolution–precipitation mechanism of carbon in nickel at high temperatures and removing the metal after the deposition process. Here carbon is introduced into Ni by deposition of C/Ni sandwich layers or by ion implantation [28] or dissolution of carbon into nickel in a plasma-enhanced CVD process [29]. Upon heating up to about 1000 °C carbon dissolves in Ni with a substantial solubility and during cooling down it segregates to the Ni–substrate interface where it precipitates as graphene. After etching off the nickel, the graphene film is exposed. The complete process sequence is shown in Figure 2 [29].

By choosing the appropriate temperature profile and C/Ni ratio the number of graphene layers can be controlled. However, process control is difficult and Ni grain boundaries can lead to an inhomogeneous thickness distribution of the graphene layer. A similar approach utilizes the diffusion of carbon species from a CVD atmosphere along grain boundaries through a copper film to the underlying substrate [30]. As there is no significant solubility of carbon in copper, the film formation is expected to be very inhomogeneous. Alternatively Cu is used as catalytic material to convert a self-assembled monolayer as carbon source at the Cu/SiO_2_ substrate to graphene [31].

A newer procedure is the use of a suitable growth substrate that is compatible with device fabrication. Such a suitable substrate material is germanium [32–33] but only a limited choice of electronic devices can make use of Ge. Thus the transfer of graphene from this growth substrate, preferably using etch-free methods due to high substrate cost [32], is an interesting option.

### 2 Intrinsic properties

The quality of the graphene material itself is a result of the growth and transfer process and can be influenced by several intrinsic properties.

#### 2.1 Grain boundaries

One property is the density of defects. In the case of good quality graphene it is mainly determined by the density of grain boundaries between the single-crystalline domains [34]. If a grain boundary lies inside the active region of a graphene device it reduces the device performance by locally changing the charge carrier mobility. Several studies have been conducted on this topic [35–36], but the impact on manufacturability is still not very clear. However, there is continuous improvement towards large crystallite sizes in the millimeter range [37–38].

#### 2.2 Contamination

Another important intrinsic property of graphene is the amount of contaminations from the synthesis and transfer process. The synthesis process mainly introduces metallic contaminations typically in the range of 10^13^ to 10^14^ atoms·cm^−2^, which corresponds to every tenth to hundredth atom in a monolayer. The main contamination is Cu from the CVD process, but also Fe is found in remarkable amounts. Several cleaning processes have been evaluated, but no substantial contamination removal could be achieved [39]. These metal contamination levels do not only lead to difficult integration into CMOS process lines (the typical upper control limit is in the range of 10^10^ to 10^11^ atoms·cm^−1^, depending on the metal type and technology). They also are influencing the graphene properties by charge-transfer doping [40–41]. The second main contamination source, polymer residues, typically originates in the transfer process from incompletely removed supporting polymer layers such as PMMA. Though, these residues usually can be removed by an appropriate vacuum annealling [42].

### 3 Substrate interactions

**Figure 3:**
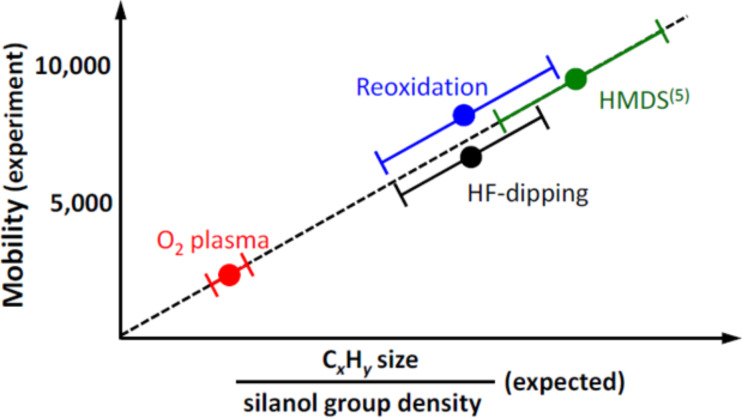
Schematic illustration of the mobility (experiment) as a function of the ratio between C*_x_*H*_y_* size and silanol group density (expected). Reprinted with permission from [45], copyright 2011 AIP Publishing.

Because graphene consists only of surface, every interaction with its environment changes its properties. Hence, also the dielectric device substrate on which a graphene layer is deposited has an influence on properties of graphene such as charge carrier mobility and density. There is a large spread of reported charge carrier mobilities for graphene on different substrates, such as 4,400–17,000 cm^2^·V^−1^·s^−1^ for SiO_2_ [43] in contrast to 25,000–140,000 cm^2^·V^−1^·s^−1^ for h-BN [44]. Besides the formation of chemical bonds, mainly two mechanisms describe the graphene–substrate interactions, charge-transfer doping and the introduction of strain on the nanoscale. Also, the surface functionalization, e.g., of SiO_2_ with hexamethyldisilazane (HMDS) has a strong influence on the charge carrier mobility (Figure 3) [45].

#### 3.1 Doping

Charges in a dielectric substrate can be introduced by foreign atoms and dangling bonds as well as by mobile ions. The doping of supported graphene samples on different substrates is influenced through these charges due to image-charge formation. SiO_2_ and h-BN shows hole doping in graphene in the range of 10^12^ to 10^13^ cm^−2^ [46–47], surface-functionalized SiO_2_ with HMDS shows a lower hole doping of approximately 1.25 × 10^12^ cm^−2^ [48]. Pre-treatment of the surface has a large influence on doping and therefore on the mobility in graphene sheets. Plasma-treated SiO_2_ surfaces reveal reduced hole doping because of the absence of organic residues, HF-treated SiO_2_ shows higher hole doping values due to fluorine dangling bonds. Also water and hydrocarbon molecules adsorbed on the SiO_2_ surface show a significant effect [45].

#### 3.2 Nanostrain

Another type of substrate–graphene interaction is the introduction of local strain induced by substrate roughness on the nanometer scale. The resulting deformation of graphene leads to degradation of the charge carrier mobility. Graphene on flat surfaces like h-BN and SiO_2_ show a compressive strain of approximately −0.1% [49]. On epitaxial germanium(001) with a higher surface roughness there is a higher compressive strain in the range between −0.37% and −0.25% [33]. Thus, it is crucial to provide substrates as smooth as possible for graphene integration.

### 4 Encapsulation

**Figure 4:**
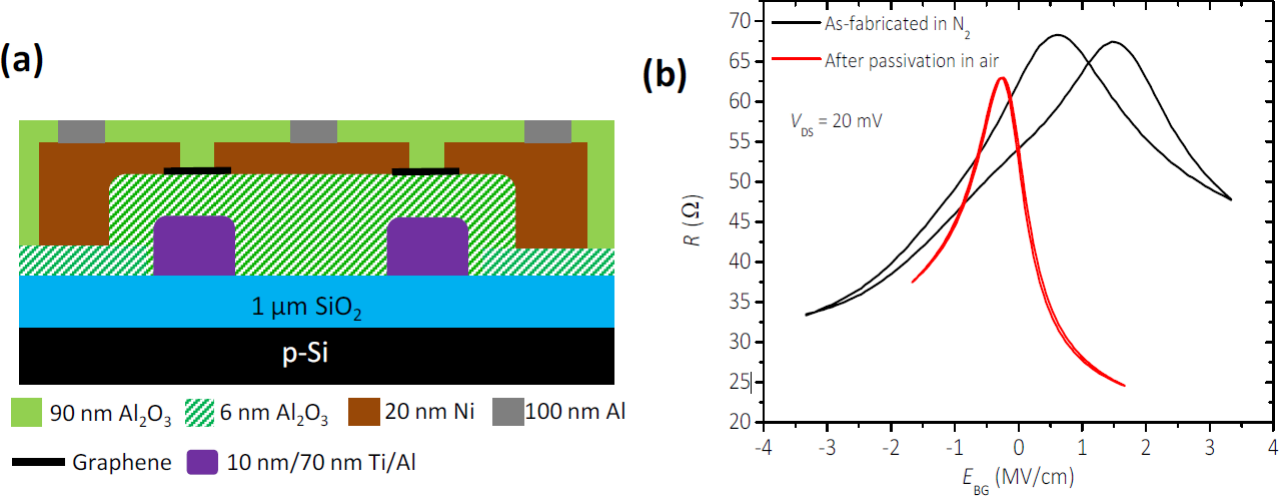
(a) Schematic showing the layers of material used in fabrication of a back-gated graphene RF-transistor. (b) Transfer function of the transistor before and after passivation. Reproduced with permission from [50], copyright 2015 The Royal Society of Chemistry.

In order to protect the graphene layer from environmental influences during further device processing an encapsulation with protective material is required. As mentioned above, the interaction of graphene with neighboring materials has to be minimized. So, a suitable material must fulfill the requirement of acting as a diffusion barrier against humidity, chemicals and gases during further processing. In addition, it has to be deposited using a process with minimal influence on graphene, e.g., high-temperature or plasma-CVD processes are to avoided. Encapsulation with exfoliated single-crystalline h-BN layers yields excellent results [4], but this is not a production-relevant approach. A material that is available in mass-production quantities and fulfills both mentioned requirements is aluminum oxide deposited by atomic-layer deposition (ALD). It was demonstrated that graphene encapsulated in Al_2_O_3_ could be passivated (Figure 4) and was stable for a longer period of time under ambient conditions [50].

The main problem of growing Al_2_O_3_ by ALD is to obtain a continuous nucleation layer on graphene to start the deposition. To solve this problem several approaches are proposed. One solution is the deposition of a few nanometers thin Al layer on graphene and subsequently oxidizing it in air to generate a thin start layer for a subsequent ALD process depositing several tens of nanometers of Al_2_O_3_ [50]. Further there is an adapted nucleation process using water and trimethylaluminium (TMA) as precursors at untypically low temperatures down to 80 °C [51].

**Figure 5:**
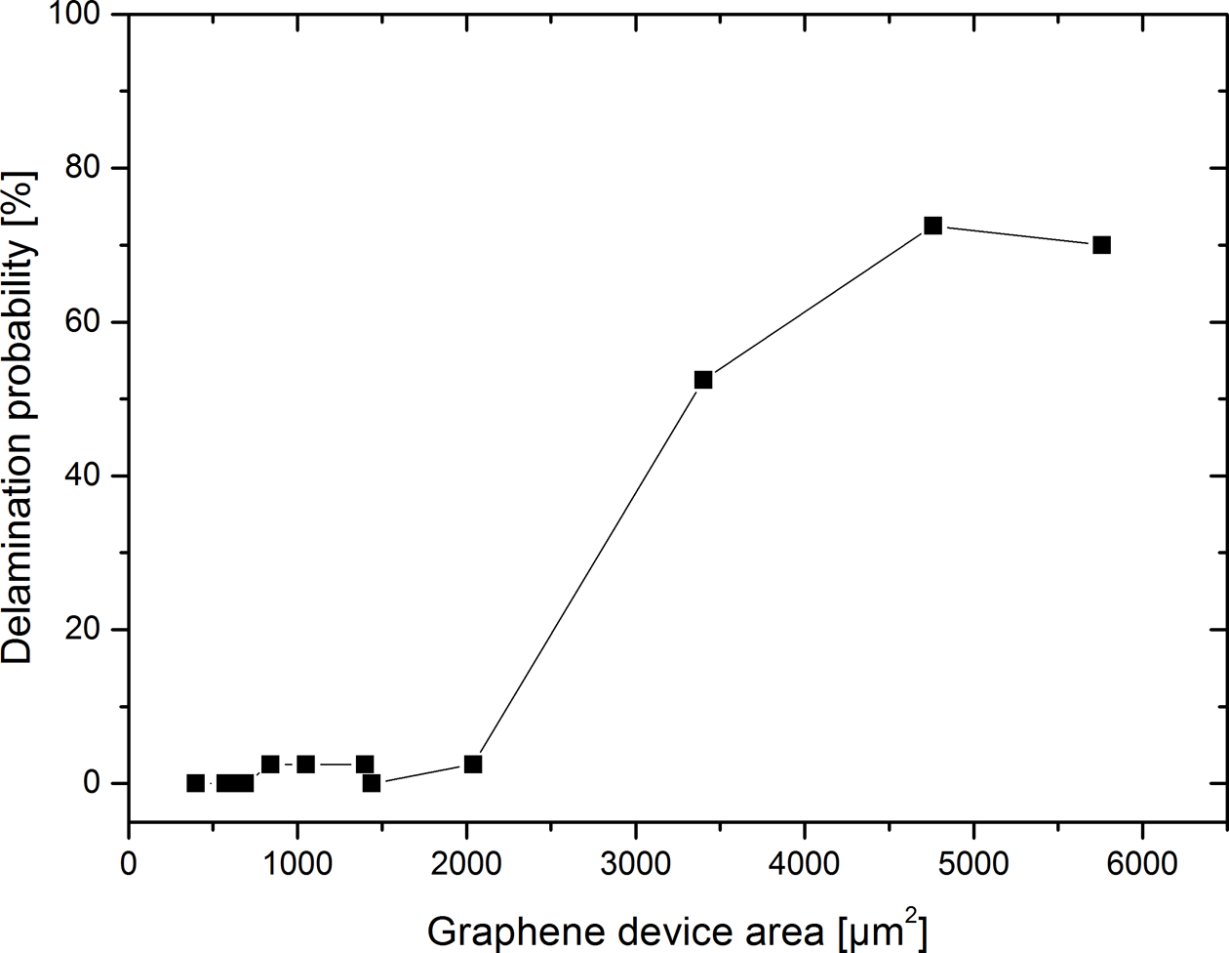
Probability of delamination of a high-*k* dielectric/Ni stack on graphene devices as a function of the device area. The data are extracted from 520 devices in total.

As little as possible interaction of graphene with the encapsulation materials is required to maintain the properties of graphene. In other words, this interaction is detrimental to the adhesion of graphene on the substrate and the encapsulation materials. The integrity of graphene devices is strongly affected by the adhesion strength. Adhesion strength generally depends on adhesion energy, device area and externally induced strain, e.g., from metal and dielectric layers. Figure 5 depicts our experimental findings on the probability of delamination of a high-*k* dielectric/nickel stack on graphene as a function of the graphene device area. The 200 nm thick Ni film was introduced as a layer to provoke delamination through tensile stress. The existence of a distinctive device area threshold for delamination can clearly be seen. This indicates that delamination effects should be manageable with respect to device manufacturing.

### 5 Contacts

**Figure 6:**
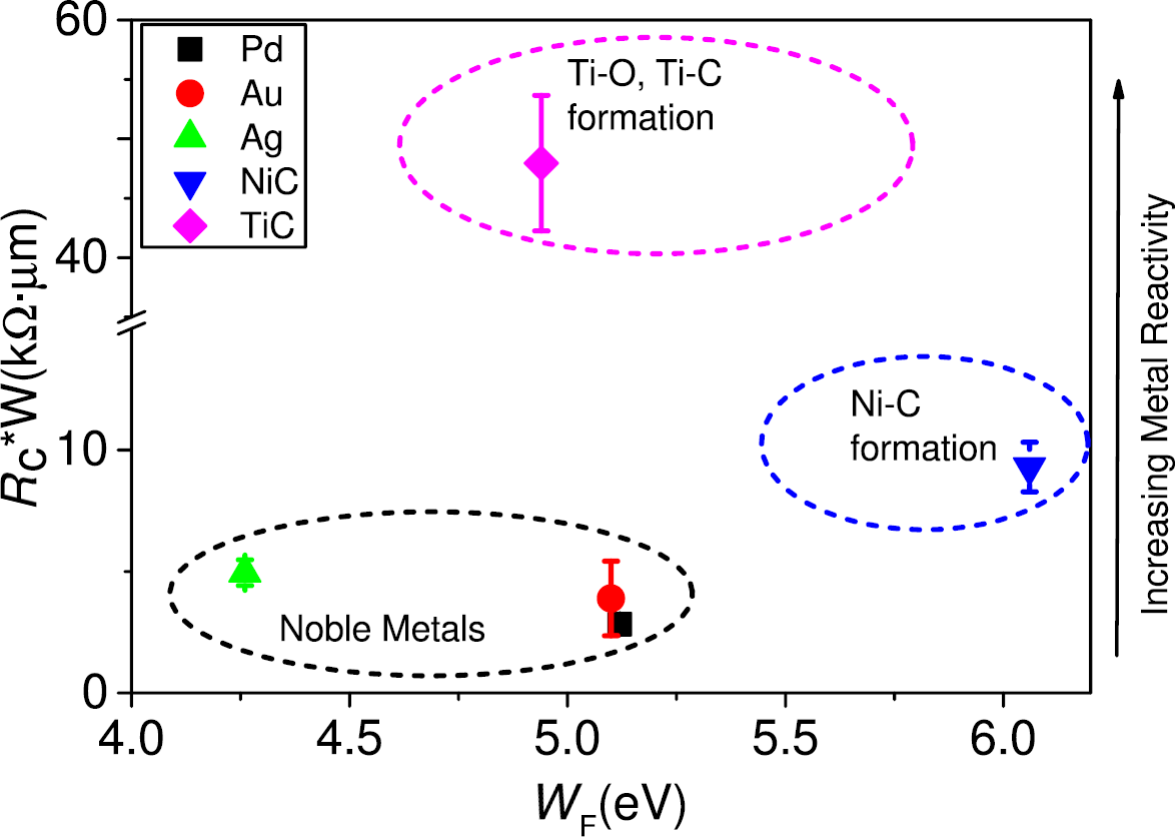
Obtained *R*_c_·contact width values as a function of the contact-metal work function (*W*_F_) and increasing metal reactivity. A clear trend can be observed correlating the high measured *R*_c_ values with the most reactive metals (Ni, Ti). Reprinted with permission from [52], copyright 2015 AIP Publishing.

Because the performance of an electronic device, e.g., a transistor, is strongly affected by parasitic effects such as contact resistances (the contact resistance should not exceed 10% of the channel resistance), this issue is of enormous importance, especially for short-channel devices. Numerous studies have been published during the last few years on the understanding and improvement of graphene–metal contacts, reporting contact resistivity values in a wide range from several ohm-micrometers to several hundred thousand ohm-micrometers. Metal contacts can interact with graphene in different ways [52], as shown in Figure 6.

**Figure 7:**
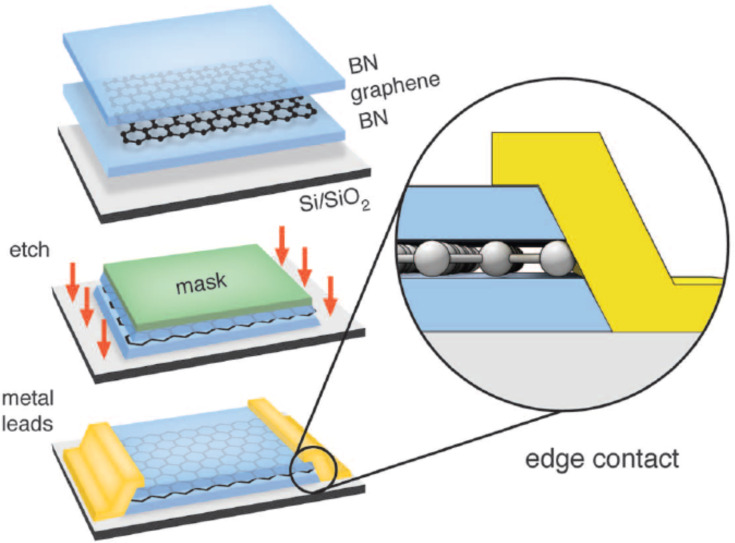
Schematic of the edge-contact fabrication process. Reprinted with permission from [58], copyright 2013 AAAS.

Metals physisorbed on graphene cause charge-transfer-induced doping of the graphene sheet because of the difference in work function values [53]. Metals chemisorbed on graphene are open a band gap in graphene. Both mechanisms result an increased contact resistance. Besides through choosing an appropriate metal, the contact resistance can be improved by thorough interface engineering [54] and applying an electrical field under the contacts to adjust the Fermi energy [55–56]. With respect to CMOS integration it seems that even CMOS-compatible contact metals such as Ni are yielding reasonable contact resistances [57]. The most promising contact-geometry approach is the formation of a one-dimensional contact, contacting the graphene film from the side [58–59] according to Figure 7. With this configuration contact resistances as low as 100 Ω·μm can be achieved.

### 6 General integration issues

**Figure 8:**
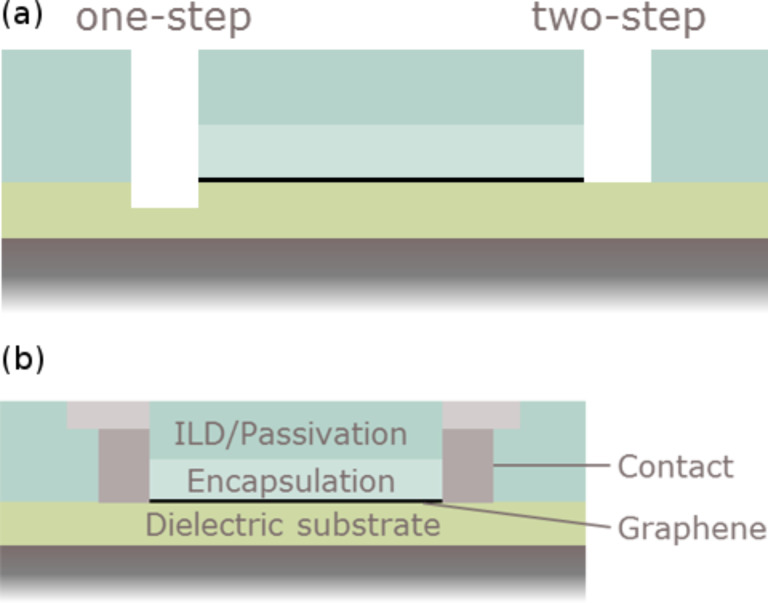
(a) Schematic of the final side-contact hole cross-section after a one-step non-selective etch process and a two-step selective etch process. (b) General schematic of the integrated device comprising side contacts.

Referring to the aspects mentioned above, the currently most suitable integration scheme includes the utilization of the encapsulation layer as etching hardmask for structuring the graphene layer in order to avoid a contamination of graphene by following lithography steps. One method utilizes a one-step plasma-etch process with low selectivity to the underlying dielectric substrate to etch the encapsulation/hardmask layer and the underlying graphene simultaneously. An alternative approach applies two consecutive steps to etch the hardmask by a selective plasma etch or wet etch, followed by a second highly substrate-selective oxygen-plasma etch step to remove graphene. Both methods are schematically depicted in Figure 8a. The first method seems to be more suitable for the fabrication of side contacts. However, it needs to be ensured that the etch polymer residues are removed thoroughly to enable optimal contact formation. Of course lateral underetch effects must be avoided. In the next step the contact metal is deposited and structured with standard processes followed by inter-layer dielectric and passivation layers. The general device schematic is depicted in Figure 8b.

Because graphene as a carbon allotrope can react with other materials at high temperatures forming, e.g., metal carbides or carbon dioxide, care has to be taken that the subsequent processing steps do not employ temperatures exceeding 500 °C. The favorite solution to this issue is the so-called graphene-last approach in which graphene devices are formed in the metallization levels of a CMOS device [60].

## Conclusion

The integration of graphene into CMOS-compatible electronic devices poses a lot of challenges when mass-production aspects are taken into account. An appropriate graphene quality can be produced by CVD on Cu substrates. Unsolved issues are the influence of grain boundaries and the reduction of metallic contamination. However, the synthesized graphene films have to be transferred to a dielectric substrate forming the device. This transfer process is critical as it is sensitive to defects, such as particles, cracks and wrinkles that degrade the graphene quality. Direct deposition methods on dielectric substrates exist, but do not yield a sufficient quality yet. We assume that this improvement of the deposition complex is crucial for commercializing graphene for microelectronics. Additionally, substrate interactions are strongly degrading the graphene quality. Hexagonal boron nitride has been identified as the ideal substrate material, but it is not available yet in the required quality for producing wafers. In order to obtain manufacturable graphene devices in the near future, established materials, such as SiO_2_, have to be adapted, even if the best possible graphene performance cannot be achieved. The encapsulation of graphene by Al_2_O_3_ seems to be solvable in the near future, as well as the formation of low-resistance graphene metal contacts. From today’s perspective the main issues for wafer-scale integration of graphene are well known, and the future of graphene (and other two-dimensional materials, namely transition metal dichalkogenides) in microelectronics depends on the successful solution of these problems.
